# Attenuated dietary motivation in Wilson’s disease correlates with clinical severity

**DOI:** 10.1186/s13023-026-04263-z

**Published:** 2026-04-03

**Authors:** Xue-Qiao Wang, Yan-Xin Wang, Zhao-Hao Ye, Zhi-Lin Zhang, Xun Pan, Xi-Chen Wang, Si-Fan Cen

**Affiliations:** Department of Neurology, The First Affiliated Hospital of Anhui University of Chinese Medicine, No. 117 Meishan Road, Hefei, Anhui 230031 China

**Keywords:** Wilson’s disease, Eating psychology, Dutch eating behavior questionnaire, Low-copper diet, PWR, Reward motivation

## Abstract

**Background & Aims:**

Wilson's disease (WD) necessitates lifelong copper-chelation therapy and a restrictive low-copper diet. While the psychological impact of dietary restriction is well-documented in obesity and diabetes, the distinctive eating psychology of WD patients remains unexplored. We aimed to characterize the eating psychology of WD patients and its association with disease severity.

**Methods:**

In a cross-sectional study, 50 WD inpatients and 110 HCs were assessed using the Food Cravings Questionnaire-Trait (FCQ-T) and the Dutch Eating Behavior Questionnaire (DEBQ). WD patients were stratified by liver function, neurological symptoms, and platelet-to-white blood cell ratio (PWR), and prevalent ATP7B genotype (R778L). Group comparisons used non-parametric tests.

**Results:**

Compared to HCs, WD patients showed significantly lower scores on all DEBQ dimensions and key FCQ-T facets (all p<0.05). Within the WD cohort, patients with milder liver disease (CTP-A) or no neurological symptoms had significantly higher scores on emotion-driven craving and food-related reinforcement than those with advanced disease (CTP-B/C) or neurological involvement (p<0.05). A low PWR, indicative of severe liver fibrosis, was associated with significantly reduced "Thoughts about food" (U=396.5, p=0.028). Exploratory analysis based on the R778L mutation revealed no significant association between genotype and dietary psychological scores.

**Conclusions:**

WD is associated with a generally attenuated dietary motivation; however, patients with less severe disease exhibit a characteristic psychological profile with relatively stronger reward-related eating motivations compared to their more severely affected counterparts. These findings highlight the need for integrated psychometric assessment in WD management to guide personalized dietary interventions and improve outcomes.

**Supplementary Information:**

The online version contains supplementary material available at 10.1186/s13023-026-04263-z.

## Introduction

Wilson’s disease (WD) is a rare autosomal recessive inherited disorder of copper metabolism, with an estimated prevalence of 1/30,000–1/50,000. The causative gene, *ATP7B*, encodes a copper-transporting P-type ATPase, which is involved in the incorporation of copper into ceruloplasmin and promotes the biliary excretion of copper from hepatocytes, thereby maintaining systemic copper homeostasis [1]. Mutations in the *ATP7B* gene lead to dysregulated copper metabolism, resulting in copper accumulation in various organs and tissues, including the liver, brain, cornea, and kidneys. This subsequently manifests as hepatic impairment, neurological symptoms, and psychiatric abnormalities. Correcting copper dysregulation is a cornerstone of WD management; hence, dietary copper restriction has long been considered an important adjunct strategy. Clinical guidelines from the American Association for the Study of Liver Diseases (AASLD) and the European Association for the Study of the Liver (EASL) recommend that patients avoid high-copper foods and water, particularly during the initial phase of decoppering treatments [2]. Standard treatment for WD currently involves the use of copper chelators and zinc salts, combined with a low-copper diet. However, long-term strict dietary restrictions may lead to insufficient intake of certain nutrients, potentially impacting patients’ quality of life [3].

In recent years, the relationship between diet and mental health has garnered increasing attention. Multiple studies indicate a significant association between diet quality and mental health status [4, 5]. For instance, a healthy diet may exert a protective effect against depression [6]. A cross-sectional study demonstrated that favorable mental health indicators (self-rated health, body satisfaction, life satisfaction, and eight symptoms) were significantly associated with breakfast quality and overall dietary patterns [7].

Although pharmacotherapy is the cornerstone of treatment for WD, patients are required to adhere to a lifelong low-copper diet. This persistent dietary restriction, intertwined with the neuropsychiatric symptoms commonly associated with WD—such as depression, apathy, and impulse control disorders—may collectively shape patients’ eating-related psychological behaviors, thereby exerting profound effects on their nutritional status and psychological adaptation. However, research on the psychological eating characteristics of WD patients and their relationship with disease features, such as liver function severity assessed by the Child-Turcotte-Pugh (CTP) classification and clinical symptom subgroups, remains limited. Therefore, this study aims to systematically investigate the differences in eating psychological patterns between WD patients and healthy individuals, and to analyze their associations with the severity of liver dysfunction and clinical symptom profiles, thereby providing a basis for developing more targeted comprehensive management strategies.

## Materials and methods

### Patient and control recruitment

This cross-sectional study enrolled 50 inpatients with WD from The First Affiliated Hospital of Anhui University of Chinese Medicine between January 2025 and September 2025. A total of 110 healthy volunteers were recruited through community advertisements as the healthy control (HC) group. The hepatic phenotype was strictly defined as the presence of clinical or biochemical evidence of liver involvement (e.g., elevated transaminases, hyperbilirubinemia, coagulopathy, or imaging findings of chronic liver disease) without dominant or debilitating neurological symptoms at the time of enrollment.

### Diagnostic criteria

#### Diagnostic criteria for WD

The diagnosis of WD was established for all patients in strict accordance with the Chinese guidelines for the diagnosis and treatment of Wilson’s disease (2022 edition). The diagnosis was primarily based on a combination of clinical features and laboratory parameters, including: age of onset (5–35 years), clinical symptoms (hepatic and/or neuropsychiatric manifestations), and copper metabolism markers (serum ceruloplasmin < 200 mg/L, 24-hour urinary copper excretion ≥ 100 μg/24 h, or hepatic copper content > 250 μg/g dry weight). The presence of Kayser-Fleischer rings on slit-lamp examination, a positive family history, and positive mutation analysis were also considered. The Leipzig score [8] was referenced to confirm the diagnosis. Specifically, patients with concomitant extrapyramidal/hepatic symptoms, positive Kayser-Fleischer rings, serum ceruloplasmin level < 200 mg/L, and 24-hour urinary copper excretion ≥ 100 μg/24 h were diagnosed with WD.

#### Inclusion and exclusion criteria

Inclusion criteria for enrolled WD patients included: (1) having received and maintained a standardized management regimen for at least one year, which included: (i) a strict low‑copper diet; (ii) combination copper‑chelating therapy with oral dimercaptosuccinic acid (DMSA) plus zinc gluconate; and (iii) physician‑led nutrition education on a balanced diet, advising limitation of saturated fat and free sugar intake; (2) predominant hepatic phenotype, defined as clinical and/or biochemical evidence of liver involvement (e.g., elevated aminotransferases, hyperbilirubinemia, coagulopathy, or imaging findings of chronic liver disease) in the absence of dominant or debilitating neurological symptoms; (3) aged between 15 and 50 years, and right-handed; (4) without severe cognitive impairment, as confirmed by a Mini-Mental State Examination (MMSE) score of > 24 performed as part of the routine neurological admission assessment, and able to comprehend the study content and complete the questionnaires.

HCs were required to meet the following criteria: (1) no history of major physical or psychiatric illnesses; (2) aged between 18 and 50 years, and right-handed.

All participants were excluded from the study if they met any of the following criteria: (1) predominant neurological or mixed (hepatic and neurological) phenotype, characterized by the presence of clinically significant neurological signs that impair daily activities, such as severe tremor, dystonia, dysarthria, or gait disturbance; (2) history of head trauma with loss of consciousness for >5 minutes, history of drug abuse or alcoholism, or pregnancy; (3) acute hepatic decompensation requiring hospitalization, severe neurological symptoms, or severe psychiatric symptoms within the past 3 months; (4) comorbid with other severe/unstable systemic diseases (e.g., uncontrolled diabetes, heart failure, terminal cancer, etc.) or other liver diseases (e.g., alcoholic, viral, autoimmune, drug-induced, or parasitic hepatitis); (5) severe visual or hearing impairment preventing effective communication; currently on a specific diet regimen (e.g., weight-loss diet, vegan diet, etc.) for any reason; (6) history of diagnosed eating disorders; (7) refusal to participate.

### Ethical considerations

All participants were fully informed of the study details prior to enrollment. Participants were asked to complete the provided questionnaires, and all information collected was kept strictly confidential and used solely for the purposes of this research. The study was approved by the Ethics Committee of The First Affiliated Hospital of Anhui University of Chinese Medicine (Approval No. 2025AH-247) and was conducted in accordance with the ethical principles of the Declaration of Helsinki. All participants provided written informed consent.

### Research instruments

#### Food craving Questionnaire-Trait

The Food Craving Questionnaire-Trait (FCQ-T), developed by Cepeda-Benito et al. and adapted into Chinese, was used to assess patients’ long-term and stable tendency for food craving [9]. This scale contains a total of 39 items, rated on a 6-point Likert scale (1=“never” to 6=“always”). It comprises seven dimensions: intentional planning for eating, positive reinforcement from eating, negative reinforcement from eating, lack of control over eating, thoughts or preoccupation with food, emotional craving, and craving triggered by environmental cues. The score for each dimension is calculated as the mean score of its respective items, ranging from 1 to 6. A higher total score indicates a more pronounced and persistent food craving trait.

#### Dutch eating behaviour questionnaire

The Dutch Eating Behaviour Questionnaire (DEBQ), developed by Van Strien et al. and widely used in eating behaviour research, was employed to evaluate the characteristics of patients’ eating behaviour [10]. The DEBQ has demonstrated high internal consistency (Cronbach’s α = 0.964) in a study of Chinese adolescents [11]. This questionnaire consists of 33 items, rated on a 5-point Likert scale (1=“never” to 5=“always”). It includes three dimensions: Restrained Eating, Emotional Eating, and External Eating. The score for each dimension is calculated as the mean score of the corresponding items, with higher scores reflecting a more prominent pattern of the respective eating behaviour.

### Data collection procedure

Data on sociodemographic and clinical characteristics, including confirmation of adherence to the standardized management regimen, were collected through face-to-face interviews and medical record reviews. Internationally recognized scales, including the FCQ-T and the DEBQ, were used in this study to assess participants’ psychological characteristics related to eating. The following sociodemographic and clinical characteristics of the participants were obtained from survey data and medical records: a) demographic characteristics (e.g., gender, age); b) lifestyle factors (e.g., smoking, alcohol consumption); c) disease-related information (e.g., family history); d) treatment details (e.g., medication use, follow-up); and e) laboratory parameters (e.g., complete blood count, coagulation profile, liver function tests, and copper metabolism indicators). All scales were self-administered by participants under the supervision of uniformly trained researchers in a quiet and independent environment. The researchers provided standardized instructions but did not interfere with the participants’ responses. Patients with WD were assessed during a stable phase of their condition in the hospital, while HCs completed the assessments at community sites. This approach ensured the standardization and consistency of data collection.

Additionally, this study utilized the CTP score/class to assess patients’ liver function. The CTP score was calculated based on prothrombin time, albumin, bilirubin, and clinical findings of ascites and encephalopathy, and it was categorized into three classes: class A (5–6), class B (7–9), and class C (10–15) [12]. The platelet-to-white blood cell ratio (PWR) has been established as an independent risk factor for hepatic complications and a robust predictor of hepatic decompensation in patients with WD [13]. Based on the cutoff value from that study, patients with a PWR ≤ 26.3 were classified into the low-PWR group, while those with values above this threshold were assigned to the high-PWR group, in order to further explore its association with dietary and psychological factors. Additionally, to evaluate the efficacy of home-based oral copper-chelation therapy and long-term treatment adherence in WD patients, we stratified participants according to their 24-hour urinary copper excretion levels measured at admission. Using a previously established cutoff [14], patients with 24-hour urinary copper excretion ≥ 465.75 μg were categorized into the high urinary copper excretion group, suggesting potentially insufficient recent decoppering therapy or poor adherence, whereas those below this value were classified into the low urinary copper excretion group. To further characterize the study cohort, based on current medical history data, WD patients were categorized into four groups according to their neurological symptoms: asymptomatic group, focal/unilateral symptom group, bilateral asymmetric symptom group, and bilateral symmetric symptom group.

## Statistical analysis

Data analysis was performed using the Statistical Package for the Social Sciences (SPSS, version 26.0). Categorical variables are presented as frequencies and percentages. Continuous variables are summarized as mean ± standard deviation (SD) if normally distributed, or as median (interquartile range) if non-normally distributed. Comparisons between groups for normally distributed continuous variables were conducted using the Independent Samples t-test. Comparisons between groups for categorical variables were conducted using the Chi-square test or Fisher’s exact test (if expected cell frequencies were less than 5). For comparisons of continuous variables between two groups, the Mann-Whitney U test was used. For comparisons across more than two groups, the Kruskal-Wallis test was applied, followed by post-hoc pairwise comparisons using Dunn’s test with Bonferroni correction for *p*-value adjustment. A two-sided *p*-value < 0.05 was considered statistically significant.

## Results

### Baseline clinical characteristics of the study cohort

The study enrolled 50 inpatients with hepatic-predominant Wilson’s disease and 110 age- and sex-matched healthy controls (HCs). The WD cohort was specifically selected to have clinical or biochemical evidence of liver involvement without dominant neurological symptoms at enrollment (see Methods 2.2.2). As summarized in Table 1, the two groups were well-matched for age, sex, and BMI (all *p* > 0.05). Reflecting their hepatic involvement, WD patients exhibited significant laboratory abnormalities, including elevated liver enzymes (ALT, AST), total bilirubin, and decreased platelet counts compared to HCs (all *p* < 0.05). The severity of liver disease, assessed by the Child-Turcotte-Pugh (CTP) classification, was distributed as follows: 35 patients (70%) were in CTP class A (compensated, mild), 12 (24%) in class B (moderate), and 3 (6%) in class C (severe). All WD patients were on standardized decoppering therapy (DMSA and zinc gluconate) with a median treatment duration of 4.50 years (IQR: 3.00, 6.00). This cohort composition allows for a focused investigation of dietary psychology in relation to hepatic severity in WD.

**Table 1:** Baseline characteristics of the study participants

Characteristic	Wilson’s Disease Group (*n* = 50)	Healthy Control Group (*n* = 110)	p-value
Demographics			
Age, years, mean ± SD	31.06 ± 11.06	32.10 ± 10.71	0.844
Male, n (%)	27.00(54.00%)	62.00(56.00%)	0.864
BMI, kg/m^2^, mean ± SD	25.20 ± 1.43	24.90 ± 1.57	0.209
WD Specific & Liver Severity			
Disease Duration, years, median (IQR)	4.50(3.00,6.00)	N/A	N/A
CTP Class A/B/C, n (%)	35.00(70.00%)/12.00(24.00%)/3.00(6.00%)	N/A	N/A
Laboratory Parameters			
Platelet count, ×10^9^/L, mean ± SD	143.42 ± 73.98	107.34 ± 12.45	0.006
Alanine Aminotransferase (ALT), U/L, median (IQR)	24.00(16.85,51.60)	21.60(14.33,36.80)	0.000
Aspartate Aminotransferase (AST), U/L, median (IQR)	27.05(20.55,36.52)	22.57(18.45,28.69)	0.002
Total Bilirubin, μmol/L, median (IQR)	15.26(10.63,23.12)	12.79(8.32,15.62)	0.019
Albumin, g/L, mean ± SD	40.92 ± 5.77	42.36 ± 3.42	0.237
Serum Ceruloplasmin, mg/L, mean ± SD	0.06 ± 0.04	N/A	N/A
24-h Urinary Copper, μg/24 h, median (IQR)	827.82(568.69,1158.28)	N/A	N/A

### Reduced eating behavior and food craving scores in WD patients

In our study, the Cronbach’s α for each dimension of the scales is presented in Supplementary Table S1. The two groups were well-matched for key demographic variables, including age, sex, and BMI (all *p* > 0.05, Table 1), indicating good comparability. WD patients scored significantly lower than the HCs on the DEBQ total score and all its subscales, including restrained eating, emotional eating, and external eating. Furthermore, WD patients also exhibited significantly lower scores on the FCQ-T total score and its specific dimensions of food craving, such as thoughts about food, emotion-induced craving, and cue-dependent craving. (Table 2).

**Table 2:** Comparison of dietary-psychological scores between patients with WD and HCs.

Measure	WD Group Median (IQR)	HC Group Median (IQR)	Mann-Whitney U	Z	*p*-value(2-tailed)
FCQ-T Total Score	108.50(93.50,137.25)	126.00(101.75,155.25)	3446.00	2.56	0.010
DEBQ Total Score	69.50(58.75,90.25)	101.00(93.75,127.25)	4579.50	6.73	0.000
Restrained Eating	20.00(14.00,27.75)	32.00(27.00,37.00)	4296.50	5.69	0.000
Emotional Eating	25.00(16.75,32.50)	42.50(36.00,49.00)	4625.50	6.90	0.000
External Eating	27.00(22.00,34.25)	32.00(27.00,40.25)	3745.50	3.66	0.000
Planning Eating	13.00(10.00,17.25)	16.00(10.00,21.00)	3199.00	1.65	0.098
Positive Reinforcement from Eating	16.00(12.00,21.00)	17.50(13.00,24.00)	3201.00	1.66	0.096
Negative Reinforcement from Eating	21.00(17,25.25.00)	19.00(14.00,25.00)	2421.00	−1.21	0.225
Lack of Control	17.00(13.00,22.00)	21.00(14.00,28.00)	3325.50	2.12	0.034
Thoughts about Food	19.00(15.00,21.00)	21.50(15.00,29.00)	3381.50	2.32	0.020
Emotion-induced Craving	15.00(1200,19.00)	21.00(16.00,26.00)	4047.00	4.78	0.000
Cue-dependent Craving	10.50(7.75,14.00)	14.00(9.00,18.00)	3522.00	2.84	0.004

### Association between CTP classification and dietary psychological characteristics

To investigate the relationship between liver disease severity and dietary motivation, WD patients were stratified into two subgroups based on CTP classification: mild disease (CTP - A) and moderate-to-severe disease (CTP - B/C). As shown in Table 3, patients with advanced liver disease (CTP-B/C) demonstrated significantly lower scores on several key dietary motivation dimensions compared to those with mild disease (CTP-A). The most pronounced differences were observed in Negative Reinforcement from Eating (*U* = 54.50, *p* < 0.001), Thoughts about Food (*U* = 68.50, *p* = 0.002), and the FCQ-T Total Score (*U* = 70.00, *p* = 0.003). Significant reductions were also found for Positive Reinforcement from Eating (*U* = 95.50, *p* = 0.023), Emotion-Induced Craving (*U* = 78.50, *p* = 0.006), and the DEBQ Total Score (*U* = 94.00, *p* = 0.021). No statistically significant differences were observed between the two subgroups in the scores for Restrained Eating, Emotional Eating, External Eating, Planning Eating, Lack of Control, or Cue-Dependent Craving (all *p* > 0.05).

**Table 3:** Dietary and psychological profiles of patients based on CTP-determined liver fibrosis severity

Measure	Subgroup A Median (IQR)	Subgroup B Median (IQR)	Mann-Whitney U	Z	*p*-value(2-tailed)
FCQ-T Total Score	118.50(99.25,138.75)	88.50(76.25,104.00)	70.00	−2.89	0.003
DEBQ Total Score	73.50(61.50,95.50)	58.50(55.75,63.75)	94.00	−2.28	0.021
Restrained Eating	21.50(15.00,30.00)	16.00(12.50,18.25)	120.00	−1.63	0.107
Emotional Eating	26.00(18.25,34.75)	18.00(13.00,22.00)	112.50	−1.82	0.069
External Eating	27.00(22.50,35.00)	24.00(19.50,26.50)	120.00	−1.63	0.107
Planning Eating	14.00(11.00,19.50)	11.50(9.50,13.00)	116.50	−1.72	0.086
Positive Reinforcement from Eating	17.00(12.25,21.75)	12.00(10.75,15.50)	95.50	−2.25	0.023
Negative Reinforcement from Eating	22.00(18.00,26.00)	13.00(12.00,19.25)	54.50	−3.29	0.000
Lack of Control	18.00(13.00,22.75)	15.50(12.75,16.00)	123.50	−1.54	0.125
Thoughts about Food	19.00(17.00,21.00)	15.00(13.50,16.25)	68.50	−2.94	0.002
Emotion-induced Craving	16.00(14.00,19.00)	12.00(10.75,14.25)	78.50	−2.68	0.006
Cue-dependent Craving	11.50(8.00,15.50)	9.00(7.75,10.25)	133.00	−1.30	0.201

### Comparison of dietary psychological characteristics based on neurological symptom severity

The Kruskal-Wallis test revealed a statistically significant difference among the four groups in the dimension of emotion-induced craving and restrained eating (Supplementary Table S2). Post hoc pairwise comparisons with Bonferroni correction showed that the asymptomatic group scored significantly higher on emotional craving than the bilateral asymmetric symptom group (adj. *p* = 0.003), and the bilateral symmetric symptom group (adj. *p* = 0.002). Although a difference was observed between the asymptomatic group and the focal/unilateral symptom group prior to correction (*p* = 0.013), it was no longer statistically significant after Bonferroni adjustment (adj. *p* = 0.082) (Supplementary Table S3). No other pairwise comparisons reached statistical significance (all adj. *p* > 0.05). For restrained eating, post hoc comparisons indicated that the asymptomatic group scored significantly higher than the bilateral symmetric group (*p* = 0.008) (Supplementary Table S4). After Bonferroni correction, the difference between the asymptomatic group and the bilateral symmetric group remained statistically significant (*p* = 0.049). The comparison between the bilateral asymmetric and asymptomatic groups was significant (adj. *p* = 0.009), but did not reach the threshold for statistical significance after Bonferroni correction (adj. *p* = 0.058). No other pairwise comparisons were significant (all adj. *p* > 0.05).

### Differences in eating-psychological indicators between subgroups

The Mann-Whitney U test revealed significant differences between the low and high PWR groups across several parameters. Specifically, the low PWR group exhibited significantly higher levels of liver fibrosis markers, including total bilirubin (*U* = 158.00, *p* = 0.009), serum hyaluronic acid (*U* = 166.00, *p* = 0.014), and type IV collagen (*U* = 189.00, *p* = 0.045) (Table 4). Conversely, scores on the food-related thoughts domain were significantly lower in the low PWR group compared to the high PWR group (*U* = 396.50, *p* = 0.028) (Table 5).

**Table 4:** Laboratory parameters in Wilson’s disease patients stratified by the PWR

Measure	Low PWR Group Median (IQR)	High PWR Group Median (IQR)	Mann-Whitney U	Z	*p*-value(2-tailed)
Red Blood Cell Count	4.36(4.16,4.95)	4.50(4.18,4.77)	279.50	−0.17	0.864
Hemoglobin	132.50(124.25,145.00)	133.00(121.50,145.00)	280.00	−0.16	0.871
Serum Albumin	41.05(32.23,44.23)	40.95(38.63,44.70)	323.50	0.71	0.473
Total Bilirubin	21.20(15.03,30.18)	13.16(10.11,19.05)	158.00	−2.62	0.009
Alanine Aminotransferase (ALT)	27.50(21.58,52.18)	21.30(16.00,46.50)	236.50	−1.04	0.298
Aspartate Aminotransferase (AST)	27.80(23.63,36.53)	25.10(19.70,36.25)	229.50	−1.18	0.237
Gamma-Glutamyl Transferase (GGT)	34.50(18.00,75.75)	36.00(17.75,64.00)	272.000	−0.32	0.746
Prothrombin Time (PT)	12.30(11.45,13.28)	11.50(10.85,12.60)	193.00	−1.92	0.054
Prolonged Prothrombin Time	−0.70(−1.55,0.28)	−1.50(−2.15,-0.40)	193.00	−1.92	0.054
International Normalized Ratio (INR)	1.05(0.97,1.14)	0.98(0.93,1.07)	199.50	−1.79	0.073
Serum Copper	2.88(2.22,5.18)	2.97(2.17,4.49)	285.00	−0.06	0.952
Serum Zinc	14.98(9.61,27.47)	19.22(13.58,20.83)	325.50	0.76	0.447
Hyaluronic Acid	155.63(106.58,211.72)	95.87(53.16,150.47)	166.00	−2.46	0.014
Type IV Collagen	74.16(58.43,78.60)	49.43(38.04,67.65)	189.00	−2.00	0.045
Ceruloplasmin	0.02(0.01,0.04)	0.04(0.02,0.05)	303.00	0.30	0.761
24-hour Urinary Copper Excretion	883.79(755.42,1184.38)	775.88(568.70,1114.28)	242.00	−0.93	0.352
24-hour Urinary Zinc Excretion	1631.96(1255.96,3599.49)	1530.48(836.17,3195.09)	257.50	−0.61	0.538

**Table 5:** Scores on dietary psychological dimensions in patients stratified by PWR

Measure	Low PWR Group Median (IQR)	High PWR Group Median (IQR)	Mann-Whitney U	Z	*p*-value(2-tailed)
Age	30.50(25.25,37.25)	26.00(20.25,34.75)	239.50	−0.98	0.327
FCQ-T Total Score	103.50(85.00,124.50)	116.50(98.75,141.25)	372.00	1.69	0.089
DEBQ Total Score	68.00(58.75,80.50)	70.50(59.75,97.25)	322.50	0.69	0.485
Restrained Eating	19.50(14.50,25.50)	20.50(14.75,32.00)	309.50	0.43	0.663
Emotional Eating	23.00(19.25,26.75)	25.50(15.25,36.25)	306.00	0.36	0.715
External Eating	27.00(22.00,32.50)	27.00(24.00,35.25)	316.50	0.57	0.564
Planning Eating	11.50(8.00,15.75)	13.50(11.75,17.25)	357.50	1.40	0.159
Positive Reinforcement from Eating	12.50(11.25,18.00)	17.00(13.00,22.25)	364.00	1.54	0.123
Negative Reinforcement from Eating	18.50(15.50,23.75)	22.00(17.75,26.00)	359.50	1.45	0.147
Lack of Control	16.00(13.00,18.00)	18.50(12.75,23.75)	343.00	1.11	0.265
Thoughts about Food	17.50(14.00,19.00)	19.00(16.75,21.00)	396.50	2.20	0.028
Emotion-induced Craving	14.50(12.00,17.75)	15.50(13.75,19.00)	348.00	1.21	0.223
Cue-dependent Craving	10.00(7.25,12.75)	11.50(8.00,16.00)	341.50	1.08	0.278

### Comparison of dietary psychological characteristics based on splenectomy status

Patients were categorized into two subgroups based on their history of splenectomy. Comparison using Mann-Whitney U tests indicated that there were no statistically significant differences between these subgroups across any of the scale dimensions (all *p* > 0.05).

### Predictive value of dietary psychological characteristics and demographic factors for high urinary copper levels at Admission

Patients were stratified into two subgroups based on whether their baseline 24-hour urinary copper excretion at admission exceeded the cutoff of 465.75 μg. Subsequent non-parametric analyses (Mann-Whitney U tests) revealed no statistically significant differences between these subgroups across any of the scale dimensions (all *p* > 0.05).

### Exploratory genotype-phenotype analysis based on *ATP7B* mutations

Patients were categorized into three groups based on their relationship to the prevalent *R778L* (c.2333G > T) mutation: *R778L* homozygotes (*n* = 14), *R778L* compound heterozygotes (carrying one *R778L* allele and another pathogenic variant, *n* = 22), and patients with other biallelic mutations (non-*R778L*, *n* = 14). Demographic, clinical severity, and dietary psychological characteristics across these three genotypic groups are summarized in Supplementary Table S5. Kruskal-Wallis tests revealed no statistically significant differences among the groups in any of the key dietary motivation scores or in indices of liver disease severity (all *p* > 0.05).

## Discussion

This study provides the first systematic characterization of dietary psychology in patients with WD, revealing a profile of generally attenuated dietary motivation compared to healthy controls. Critically, this attenuation was not uniform but varied with disease severity, being most pronounced in patients with advanced hepatic dysfunction or neurological involvement. Our findings highlight that eating behavior in WD is shaped not only by prescribed dietary restrictions but also by the underlying neuropsychiatric and hepatic pathophysiology of the disease.

Compared to HCs, WD patients exhibited significantly lower scores across all major dimensions of the DEBQ and FCQ-T, including emotional eating, external eating, and food-related thoughts (Table 2). This broad reduction suggests a global dampening of appetitive drive, contrasting with the heightened food preoccupation often seen in restrictive dieting for conditions like obesity. We propose that this hypo-responsive state may stem from copper deposition in key brain regions governing reward and motivation, such as the basal ganglia and prefrontal cortex [15]. Damage to these circuits, particularly those involving dopaminergic signaling and the melanocortin system, could impair both the hedonic and motivational aspects of food intake [16, 17]. Thus, the observed psychological profile in WD likely reflects a neurological deficit in reward processing rather than successful cognitive restraint. A methodological strength relevant to this discussion is the uniformity of decoppering therapy across our cohort. In accordance with our inclusion criteria, every patient received long-term combination therapy with DMSA and zinc gluconate, coupled with comprehensive nutritional guidance. This consistency effectively controls for a potential major confounder—differential effects of distinct therapeutic agents on appetite and eating behavior. Consequently, the associations we report between attenuated dietary motivation and markers of hepatic or neurological disease severity are less likely to be spuriously driven by variations in medical treatment.

Our subgroup analysis based on CTP classification revealed a gradient of attenuated dietary motivation, where patients with moderate-to-severe liver dysfunction (CTP-B/C) scored significantly lower on several key psychological dimensions—most notably Negative Reinforcement from Eating, Thoughts about Food, and the FCQ-T Total Score—compared to those with compensated disease (CTP-A). This finding suggests that the suppression of food-related motivation and cognition progresses in parallel with hepatic deterioration in WD, reflecting a neuro-psychobiological continuum. Several mechanisms may underlie this association. First, advanced liver disease is characterized by systemic inflammation and cytokine release (e.g., TNF-α, IL-6), which are known to suppress appetite and alter reward processing centrally [18]. In WD, copper accumulation itself may exacerbate neuroinflammation, further dampening motivational circuits [19]. Second, cirrhosis and minimal hepatic encephalopathy may impair prefrontal and limbic function, reducing cognitive engagement with food [20]. The selective reduction in reward-related dimensions (e.g., reinforcement, craving) but not in restraint suggests that the core deficit lies in the internal motivational valuation of food, which diminishes as liver function declines.

The severity of neurological symptoms was also associated with distinct dietary psychological profiles. Asymptomatic patients scored significantly higher on Emotion-Induced Craving and Restrained Eating than those with bilateral motor symptoms (Supplementary Table S2, S3, S4). This pattern may reflect a dynamic psychological adaptation process. Specifically, the higher craving and restraint scores in asymptomatic patients may be driven by an aberrant emotion-food linkage, where eating serves a maladaptive emotion regulation function [21]. This behavior can be reinforced through two pathways: negative reinforcement (temporary relief from negative affect) and positive reinforcement (pursuit of pleasure from food) [22]. The concurrent pull of these reinforcement mechanisms creates a high motivational load [22], which could explain both the heightened craving and the increased cognitive effort (restraint) required to manage it. Conversely, symptomatic patients, with likely greater fronto-striatal circuit impairment, may experience deficits in the top-down control necessary for sustained dietary restraint [15, 23], while also suffering from a more fundamental blunting of appetitive drive due to neurological damage, leading to an overall reduction in eating-related motivation. These findings suggest a dynamic psychological adaptation process across different stages of the disease. Therefore, implementing differentiated psychological and behavioral intervention strategies tailored to the specific disease stage is crucial.

Our finding of a significant association between a low PWR and reduced scores on the “Thoughts about Food” dimension (Table 5) extends the link between disease severity and dietary psychology to the realm of systemic inflammation and fibrosis. The PWR, a readily calculable and cost-effective inflammatory and immune biomarker derived from routine blood tests, has been utilized to assess disease severity and prognosis in various hepatic disorders. Our study demonstrated that a low PWR was significantly associated with the severity of liver fibrosis. Consistent with previous reports [24, 25], patients in the low-PWR group exhibited significantly higher serum levels of hyaluronic acid and type IV collagen compared to the high-PWR group, indicating more advanced liver fibrosis and injury. This finding further suggests that patients with low PWR may be at a higher risk of progressing to decompensated cirrhosis complications [13], such as splenomegaly/hypersplenism, gastroesophageal varices, and ascites. The concomitant reduction in “Thoughts about Food” in the low-PWR group can be interpreted as a cognitive manifestation of this advanced disease state. From a pathophysiological standpoint, decreased appetite is common in patients with liver disease, particularly in advanced cirrhosis. The underlying mechanisms involve elevated circulating inflammatory cytokines (e.g., TNF-α, IL-1, IL-6), leptin resistance, and neuroendocrine dysregulation, which collectively contribute to pathological anorexia [26, 27]. Consequently, patients with low PWR, reflecting a more severe inflammatory and disease state, likely experience a general reduction in hunger and natural interest in food, manifesting as diminished spontaneous cognitive activity related to food. Furthermore, severe hepatic dysfunction can lead to metabolic disturbances such as hyperammonemia, which may impair brain function [28] and further suppress fundamental drives and cognitive processes related to food-seeking and consumption. On the psychobehavioral level, our results illustrate a shift in the dominant factor governing eating behavior across the disease spectrum. For high-PWR patients, whose nutritional status is relatively preserved, long-term adherence to strict dietary restrictions (e.g., a low-copper diet) is essential for disease control. This sustained, conscious self-monitoring likely directs more cognitive resources toward food-related content [29], resulting in higher scores for “thoughts about food,” despite their restricted intake. In contrast, for low-PWR patients, who are in advanced disease stages, the dominant factor governing eating behavior shifts from active psychological management to passive physiological suppression. Physiologic anorexia, driven by inflammatory cytokines and metabolic disturbances, markedly reduces spontaneous appetite and hunger [30, 31]. In this state, the food-seeking is substantially attenuated. Therefore, these patients no longer experience the psychological conflict of “wanting to eat but needing to restrain” characteristic of high-PWR patients, leading to a natural reduction in food-related spontaneous cognitive activity [32], which is reflected in their significantly lower “thoughts about food” scores. Critically, the clinical interpretation of this low score differs fundamentally from that in healthier patients. It does not signify more successful dietary management or psychological adaptation but rather indicates that the disease has progressed to a stage where potent pathophysiological factors have overridden intrinsic appetite regulation and psychobehavioral control. Thus, within the context of WD, a low score on “Thoughts about Food” alongside a low PWR may serve as an integrative marker, signaling a transition towards a state where hepatic dysfunction itself becomes the primary modulator of eating-related cognition, with implications for both nutritional risk and prognosis.

Furthermore, the observed heterogeneity in the severity of liver fibrosis (as indicated by CTP class and PWR) and neurological involvement among our WD patients is likely attributable not only to differential copper accumulation but also to interindividual genetic susceptibility [33]. Although copper toxicity is the principal pathogenic mechanism, genetic modifiers can significantly influence disease expression. For example, variants in the *PNPLA3* gene (e.g., rs738409), which are strongly associated with hepatic steatosis and fibrosis progression in various liver diseases, may modulate both hepatic phenotype and related appetite dysregulation in WD [34]. Similarly, the apolipoprotein E (*APOE*) ε4 allele, a well-established genetic risk factor for Alzheimer’s disease and other neurodegenerative processes [35], may represent a potential genetic modifier in WD. Its influence on neuroinflammatory responses and neuronal repair mechanisms could theoretically modulate the susceptibility to, or progression of, neurological symptoms [36]. Future psychometric assessments should account for these genetic variables to isolate the direct effects of copper toxicity versus genetic susceptibility. Future psychometric assessments should account for these genetic variables to isolate the direct effects of copper toxicity versus genetic susceptibility. Although our exploratory analysis of the prevalent *R778L* mutation did not reveal significant associations with dietary psychology in this cohort, the potential role of broader genetic modifiers warrants further investigation.

From the perspective of clinical management in WD, this psychological and behavioral profile presents a significant challenge. The cornerstone of WD treatment is strict adherence to a low-copper diet, which necessitates avoiding foods high in copper such as nuts, chocolate, organ meats, and shellfish. However, patients in Subgroup A, who are more prone to emotion-driven, impulsive eating, may experience substantial difficulties in maintaining this dietary regimen. During periods of emotional distress, these patients might struggle to resist the urge to consume prohibited foods [37], potentially leading to excessive copper intake, disease exacerbation, or worsening of neurological symptoms [38]. Furthermore, this pattern of eating increases the risk of overeating and binge-eating behaviors, thereby elevating the likelihood of metabolic complications such as obesity and Metabolic Dysfunction-Associated Steatotic Liver Disease (MASLD) [39], which considerably complicates overall clinical management.

From a clinical perspective, these findings argue for an integrated biopsychosocial approach to WD management. While dietary copper restriction remains a cornerstone of therapy, our data show that patients’ ability to engage with this regimen is intrinsically linked to their disease state. Patients with milder liver disease (CTP-A) and fewer neurological symptoms retain relatively stronger reward-related eating motivations, placing them at higher risk for non-adherence during emotional distress. In contrast, patients with advanced disease may appear “compliant” due to physiological anorexia, masking a high risk of malnutrition. Therefore, the integration of routine psychometric assessments (e.g., using DEBQ/FCQ-T) into standard outpatient care for patients with WD should be considered to characterize eating-related psychological profiles and to facilitate individualized dietary interventions. For some, strategies to manage emotion-driven eating may be crucial; for others, nutritional support to counter appetite loss is a priority.

## Limitations

First, this study adopts a cross-sectional design. While this design effectively reveals associations between dietary psychological characteristics and clinical indicators in patients with WD, its inherent nature precludes any inference of causality or dynamic evolution from these data. Therefore, the observed associations should be considered preliminary evidence, and the potential causal pathways require clarification in future research.

Second, the sample size of this study, though considerable within the context of a single-center setting, remains insufficient for in-depth subgroup analyses (e.g., stratification based on the degree of hepatic fibrosis or neurological symptoms). We note that the sample sizes for certain subgroups were relatively small, which may limit the statistical power of the related analyses and potentially affect the generalizability of the results. Nonetheless, the significant patterns revealed by the current subgroup analyses provide a clear rationale for targeted validation in larger-scale cohorts.

Third, the assessment of dietary psychological characteristics primarily relied on validated self-report questionnaires, which carry inherent risks of recall and social desirability bias. Similarly, the evaluation of liver fibrosis severity depended on clinical surrogate scores (CTP and PWR) rather than direct measurement via transient elastography (FibroScan). While both approaches represent standard practice in their respective fields and provide valuable clinical data, the use of these indirect measures may collectively influence the precision of the correlations observed between psychological profiles and the severity of hepatic disease.

Fourth, while we conducted an exploratory analysis of the common *ATP7B R778L* mutation, the study lacked data on potential genetic modifiers (e.g., *PNPLA3*, *APOE*) that could influence disease heterogeneity. Investigating their role remains a task for future studies.

Finally, future studies could benefit from incorporating standardized assessments of mood disorders (e.g., anxiety and depression scales) to better delineate their specific contributions to the dietary psychological profile observed in WD.

## Conclusion

This study provides the first systematic elucidation of the distinctive dietary psychological characteristics prevalent among patients with WD. A key finding is the intrinsic relationship between these psychological features and indicators of disease severity, such as hepatic fibrosis and neurological symptoms, suggesting they extend beyond being a mere consequence of dietary restrictions. Consequently, our findings underscore the necessity for WD clinical management to transcend the conventional, singular biomedical model—focused solely on decoppering therapy and dietary copper restriction—and shift towards an integrated biopsychosocial approach. Within this model, the assessment and support of patients’ dietary psychology should be established as an essential component of standard treatment.

## Electronic supplementary material

Below is the link to the electronic supplementary material.

Supplementary material 1

Supplementary Material 2

Supplementary Material 3

Supplementary Material 4

Supplementary Material 5

Supplementary Material 6

## Data Availability

The survey datasets supporting the findings of this study are available within the paper’s Supplementary Information files. The original clinical and serum biomarker data contain sensitive patient information and are not publicly available to protect participant privacy. These data are available from the corresponding author upon reasonable request and will be subject to a data access agreement approved by the Ethics Committee of The First Affiliated Hospital of Anhui University of Chinese Medicine.

## References

[1] Shribman S, Marjot T, Sharif A, Vimalesvaran S, Ala A, Alexander G, et al. Investigation and management of Wilson’s disease: a practical guide from the British Association for the study of the liver. Lancet Gastroenterol Hepatol. 2022;7(6):560–75. 35429442 10.1016/S2468-1253(22)00004-8

[2] Saroli Palumbo C, Schilsky ML. Clinical practice guidelines in Wilson disease. Ann Transl Med. 2019;7(Suppl 2):S65. 31179302 10.21037/atm.2018.12.53 PMC6531645

[3] Teufel-Schäfer U, Forster C, Schaefer N. Low copper diet-A therapeutic option for Wilson Disease? Children (Basel). 2022;9(8). 10.3390/children9081132 PMC9406399 36010023

[4] Wang X, He X, Fu K, Zhang Y. The influence of early diet quality on the mental health of college students: the mediation effects of height and qi-deficiency. Front Public Health. 2024;12:1363866. 38655517 10.3389/fpubh.2024.1363866 PMC11037246

[5] Lustermans H, Bruinhof N, Beijers R, de Weerth C. From pregnancy to postpartum: the interplay between maternal mental health and diet. Appetite. 2025;216:108253. 40754146 10.1016/j.appet.2025.108253

[6] Ataei Kachouei A, Kamrani F, Haghighatdoost F, Mohammadifard N, Najafi F, Farshidi H, et al. Relationship of the prime diet quality score (PDQS) and healthy eating index (HEI-2015) with depression and anxiety: a cross-sectional study. BMC Public Health. 2024;24(1):2919. 39438905 10.1186/s12889-024-20369-0 PMC11494750

[7] Liu L, Guo C, Lang F, Yan Y. Association of breakfast, total diet quality, and mental health in adolescents: a cross-sectional study of HBSC in Greece. Eur J Pediatr. 2023;182(12):5385–97. 37740042 10.1007/s00431-023-05180-0

[8] EASL Clinical Practice Guidelines: Wilson’s disease. J Hepatol. 2012;56(3):671–85. 22340672 10.1016/j.jhep.2011.11.007

[9] Cepeda-Benito A, Gleaves DH, Fernández MC, Vila J, Williams TL, Reynoso J. The development and validation of Spanish versions of the state and Trait food cravings questionnaires. Behav Res Ther. 2000;38(11):1125–38. 11060941 10.1016/s0005-7967(99)00141-2

[10] Van Strien T. FJER, Bergers G.P.A., Defares P.B. :. The Dutch eating behavior Questionnaire (DEBQ) for assessment of restrained, emotional, and external eating behavior. Int J Eat Disord. 1986;5:295–315.

[11] Wu S, Cai T, Luo X. Validation of the Dutch eating behavior Questionnaire (DEBQ) in a sample of Chinese adolescents. Psychol Health Med. 2017;22(3):282–88. 27080537 10.1080/13548506.2016.1173712

[12] Pugh RN, Murray-Lyon IM, Dawson JL, Pietroni MC, Williams R. Transection of the oesophagus for bleeding oesophageal varices. Br J Surg. 1973;60(8):646–49. 4541913 10.1002/bjs.1800600817

[13] Zhong HJ, Chen JY, Wu WM, He Z XX, YQ. Clinical significance of platelet-to-white blood cell ratio in patients with Wilson disease: a retrospective cohort study. PeerJ. 2025;13:e19379. 10.7717/peerj.19379 PMC12047222 40321812

[14] Walshe JM. The pattern of urinary copper excretion and its response to treatment in patients with Wilson’s disease. Qjm. 2011;104(9):775–78. 21622540 10.1093/qjmed/hcr073

[15] Tichelaar JG, Sayalı C, Helmich RC, Cools R. Impulse control disorder in Parkinson’s disease is associated with abnormal frontal value signalling. Brain. 2023;146(9):3676–89. 37192341 10.1093/brain/awad162 PMC10473575

[16] Barnhart WR, Braden AL, Price E. Emotion regulation difficulties interact with negative, not positive, emotional eating to strengthen relationships with disordered eating: an exploratory study. Appetite. 2021;158:105038. 33186623 10.1016/j.appet.2020.105038

[17] Sköld J, Lazzarino GP, Nett M, Wiskerke J, Engblom D. Melanocortin 4 receptor-expressing neurons in the lateral stripe of the striatum regulate affect and motor control. iScience. 2025;28(5):112456. 40352729 10.1016/j.isci.2025.112456 PMC12063154

[18] Cosgrove KT, Burrows K, Avery JA, Kerr KL, DeVille DC, Aupperle RL, et al. Appetite change profiles in depression exhibit differential relationships between systemic inflammation and activity in reward and interoceptive neurocircuitry. Brain Behav Immun. 2020;83:163–71. 31604141 10.1016/j.bbi.2019.10.006 PMC6937709

[19] Yang W, Xiao C, Zheng J, Song J, Li XG. Copper homeostasis and cuproptosis represent emerging targets for therapeutic intervention in inflammatory diseases. Pharmacol Res. 2025;221:107988. 41072834 10.1016/j.phrs.2025.107988

[20] Chen HJ, Lin HL, Chen QF, Liu PF. Altered dynamic functional connectivity in the default mode network in patients with cirrhosis and minimal hepatic encephalopathy. Neuroradiology. 2017;59(9):905–14. 28707166 10.1007/s00234-017-1881-4

[21] Wen J, Inauen J, Miao M. Negative affect and emotional eating: daily dynamics and their links to difficulties in emotional regulation. Appetite. 2025;213:108049. 40349933 10.1016/j.appet.2025.108049

[22] Xu S, Sun Y, Huang M, Huang Y, Han J, Tang X, Ren W: Emotional state and feedback-related negativity induced by positive, Negative, and combined Reinforcement. Front Psychol. 2021;12:647263. 34040560 10.3389/fpsyg.2021.647263 PMC8141566

[23] Zhuang X, Lemak J, Sridhar S, Nelson AB. Aberrant striatal firing mediates impulsive decision-making in a mouse model of Parkinson’s disease. Brain. 2025. 10.1093/brain/awaf312 PMC12597312 40904197

[24] Kim JH, Kim SE, Song DS, Kim HY, Yoon EL, Kim TH, et al. Platelet-to-white blood cell ratio is associated with adverse Outcomes in cirrhotic patients with Acute deterioration. J Clin Med. 2022;11(9). 10.3390/jcm11092463 PMC9103428 35566588

[25] Jie Y, Gong J, Xiao C, Zhu S, Zhou W, Luo J, Chong Y, Hu B: Low platelet to white blood cell ratio indicates poor prognosis for Acute-on-chronic liver failure. Biomed Res Int 2018. 2018;7394904. 10.1155/2018/7394904 PMC5964479 29854786

[26] da Silva BS, Paulino AMB, Taffarel M, Borba IG, Telles LO, Lima VV, Aguiar DH, Dias MC, Nascimento AF, Sinhorin VDG et al. High sucrose diet attenuates oxidative stress, inflammation and liver injury in thioacetamide-induced liver cirrhosis. Life Sci. 2021;267:118944 10.1016/j.lfs.2020.118944 33359749

[27] Ockenga J, Tietge UJ, Böker KH, Manns MP, Brabant G, Bahr MJ. Distinct roles of free leptin, bound leptin and soluble leptin receptor during the metabolic-inflammatory response in patients with liver cirrhosis. Aliment Pharmacol Ther. 2007;25(11):1301–09. 17509098 10.1111/j.1365-2036.2007.03327.x

[28] Jin YY, Singh P, Chung HJ, Hong ST. Blood ammonia as a possible etiological agent for Alzheimer’s disease. Nutrients. 2018;10(5). 10.3390/nu10050564 PMC5986444 29734664

[29] Volz S, Ward A, Mann T. Eating up cognitive resources: does attentional consumption lead to food consumption? Appetite. 2021;162:105165. 33609586 10.1016/j.appet.2021.105165 PMC8058276

[30] Li Y, Jiang Q, Wang L. Appetite regulation of TLR4-induced inflammatory signaling. Front Endocrinol (Lausanne). 2021;12:777997. 34899611 10.3389/fendo.2021.777997 PMC8664591

[31] Martins C, Roekenes JA, Rehfeld JF, Hunter GR, Gower BA. Metabolic adaptation is associated with a greater increase in appetite following weight loss: a longitudinal study. Am J Clin Nutr. 2023;118(6):1192–201. 37863431 10.1016/j.ajcnut.2023.10.010

[32] Filippone L, Shankland R, Hallez Q. The relationships between social media exposure, food craving, cognitive impulsivity and cognitive restraint. J Eat Disord. 2022;10(1):184. 36434703 10.1186/s40337-022-00698-4 PMC9701005

[33] Pennisi G, Pipitone RM, Cammà C, Di Marco V, Di Martino V, Spatola F, et al. PNPLA3 rs738409 C>G Variant predicts fibrosis progression by noninvasive tools in Nonalcoholic Fatty liver disease. Clin Gastroenterol Hepatol. 2021;19(9):1979–81. 32898706 10.1016/j.cgh.2020.09.009

[34] Grimaudo S, Pipitone RM, Pennisi G, Celsa C, Cammà C, Di Marco V, et al. Association between PNPLA3 rs738409 C & amp;#x003E;G Variant and liver-related Outcomes in patients with Nonalcoholic Fatty liver disease. Clin Gastroenterol Hepatol. 2020;18(4):935–44.e933. 31419571 10.1016/j.cgh.2019.08.011

[35] Quan M, Wang Q, Qin W, Wang W, Li F, Zhao T, et al. Shared and unique effects of ApoEε4 and pathogenic gene mutation on cognition and imaging in preclinical familial Alzheimer’s disease. Alzheimers Res Ther. 2023;15(1):40. 36850008 10.1186/s13195-023-01192-y PMC9972804

[36] Wang S, Li B, Solomon V, Fonteh A, Rapoport SI, Bennett DA, Arvanitakis Z, Chui HC, Sullivan PM, Yassine HN: Calcium-dependent cytosolic phospholipase A(2) activation is implicated in neuroinflammation and oxidative stress associated with ApoE4. Mol Neurodegener. 2022;17(1):42. 35705959 10.1186/s13024-022-00549-5 PMC9202185

[37] Raspopow K, Abizaid A, Matheson K, Anisman H. Anticipation of a psychosocial stressor differentially influences ghrelin, cortisol and food intake among emotional and non-emotional eaters. Appetite. 2014;74:35–43. 24295926 10.1016/j.appet.2013.11.018

[38] Wu Z, Song X, Wang G, Wang B. U-shaped nonlinear relationship between dietary copper intake and peripheral neuropathy. Sci Rep. 2024;14(1):25263. 39448725 10.1038/s41598-024-76159-6 PMC11502861

[39] Casagrande M, Boncompagni I, Forte G, Guarino A, Favieri F. Emotion and overeating behavior: effects of alexithymia and emotional regulation on overweight and obesity. Eat Weight Disord. 2020;25(5):1333–45. 31473988 10.1007/s40519-019-00767-9

